# Transcript profiling of *crown rootless1 *mutant stem base reveals new elements associated with crown root development in rice

**DOI:** 10.1186/1471-2164-12-387

**Published:** 2011-08-01

**Authors:** Yoan Coudert, Martine Bès, Thi Van Anh Le, Martial Pré, Emmanuel Guiderdoni, Pascal Gantet

**Affiliations:** 1 Université Montpellier 2, UMR DAP, Place Eugène Bataillon, 34095 Montpellier Cedex 5, France; 2 CIRAD, UMR DAP, Avenue Agropolis TA A-96/03, 34398 Montpellier Cedex 5, France; 3 Université Montpellier 2, UMR DIADE, Place Eugène Bataillon, 34095 Montpellier Cedex 5, France; 4 University of Sciences and Technology of Hanoi, Department of Biotechnology and Pharmacology, Institute of Agricultural Genetics, Laboratoire Mixte International Rice Functional Genomics and Plant Biotechnology, Ha Noi, Viet Nam; 5 IRD, UMR DIADE, Avenue Agropolis, 34398 Montpellier Cedex 5, France

## Abstract

**Background:**

In rice, the major part of the post-embryonic root system is made of stem-derived roots named crown roots (CR). Among the few characterized rice mutants affected in root development, *crown rootless1 *mutant is unable to initiate crown root primordia. *CROWN ROOTLESS1 *(*CRL1*) is induced by auxin and encodes an AS2/LOB-domain transcription factor that acts upstream of the gene regulatory network controlling CR development.

**Results:**

To identify genes involved in CR development, we compared global gene expression profile in stem bases of *crl1 *mutant and wild-type (WT) plants. Our analysis revealed that 250 and 236 genes are down- and up-regulated respectively in the *crl1 *mutant. Auxin induces *CRL1 *expression and consequently it is expected that auxin also alters the expression of genes that are early regulated by CRL1. To identify genes under the early control of CRL1, we monitored the expression kinetics of a selected subset of genes, mainly chosen among those exhibiting differential expression, in *crl1 *and WT following exogenous auxin treatment. This analysis revealed that most of these genes, mainly related to hormone, water and nutrient, development and homeostasis, were likely not regulated directly by CRL1. We hypothesized that the differential expression for these genes observed in the *crl1 *mutant is likely a consequence of the absence of CR formation. Otherwise, three *CRL1-*dependent auxin-responsive genes: *FSM (FLATENNED SHOOT MERISTEM)/FAS1 (FASCIATA1), GTE4 (GENERAL TRANSCRIPTION FACTOR GROUP E4) *and *MAP *(*MICROTUBULE-ASSOCIATED PROTEIN*) were identified. *FSM/FAS1 *and *GTE4 *are known in rice and *Arabidopsis *to be involved in the maintenance of root meristem through chromatin remodelling and cell cycle regulation respectively.

**Conclusion:**

Our data showed that the differential regulation of most genes in *crl1 *versus WT may be an indirect consequence of *CRL1 *inactivation resulting from the absence of CR in the *crl1 *mutant. Nevertheless some genes, *FAS1/FSM*, *GTE4 *and *MAP*, require *CRL1 *to be induced by auxin suggesting that they are likely directly regulated by CRL1. These genes have a function related to polarized cell growth, cell cycle regulation or chromatin remodelling. This suggests that these genes are controlled by CRL1 and involved in CR initiation in rice.

## Background

Molecular mechanisms underlying initiation of new roots have been extensively studied in the plant model *Arabidopsis thaliana (Arabidopsis) *(for a recent review see [1]). In the embryonic primary root of *Arabidopsis*, new root meristems derive from pericycle founder cells. These meristems give rise to post-embryonic roots named lateral roots (LR). Several genes related to auxin, involved in the mitotic competence of pericycle cells, in LR primordia initiation, patterning and LR emergence have been identified [2-7]. In cereals, the major part of the root system is made of post-embryonic stem-derived roots named crown roots (CR). Rice (*Oryza sativa *L.) is a relevant model to study the genetic control of CR development [8]. To date only a few rice mutants with less or no crown root have been identified. One of those is altered in *CRL4 (CROWN ROOTLESS4)/OsGNOM1 *that encodes an ARF-GEF (ADP-RIBOSYLATION FACTOR-GUANIDINE EXCHANGE FACTOR) [9,10] and is homologous to *Arabidopsis AtGNOM1 *[5]. AtGNOM1 regulates the intracellular traffic of PIN1 (PINFORMED1) auxin efflux carrier proteins [11], and by consequence modulates polar auxin transport involved in cell division events leading to the differentiation of LR meristem [1]. The study of the *crl4/Osgnom1 *mutant in rice suggested that OsPIN may regulate polarised auxin transport that controls the first divisions of the ground meristem, the tissue giving birth to CR [9,10]. Two other mutants named *adventitious rootless1 (arl1) *and *crown rootless1 *(*crl1*) are devoid of crown root [12,13]. They are both affected in the *ARL1/CRL1 *gene (referred as *CRL1 *hereafter) that encodes an AS2/LOB (ASYMMETRIC LEAVES2/LATERAL ORGAN BOUNDARIES)-domain transcription factor [14]. *CRL1 *is expressed in parenchyma cells adjacent to the peripheral vascular cylinder of the stem that is the area of CR initiation [12]. *CRL1 *expression is induced by auxin likely via direct binding of an ARF (AUXIN RESPONSE FACTOR) transcription factor (TF) to its promoter [12]. *CRL1 *is homologous to *Arabidopsis LBD16 *(*LATERAL ORGAN BOUNDARIES-DOMAIN PROTEIN 16*) and *LBD29 *that are directly induced by auxin via ARF7 and ARF19 and necessary for lateral root initiation [15].

The absence of CR primordia in *crl1 *mutant suggests that the CRL1 transcription factor acts upstream of the gene regulatory network that control the early steps of CR primordia differentiation. In order to identify genes involved in this process, we compared global gene expression profiles in stem bases of *crl1 *and wild-type (WT) plants, using rice expression arrays (Affymetrix). We identified 486 genes differentially expressed in the *crl1 *mutant. To arrange molecular events downstream of auxin and *CRL1*, we analysed expression kinetics of a selected subset of 47 genes in response to auxin treatment in *crl1 *and WT. This allowed to identify 3 *CRL1-*dependent auxin responsive genes. Two of them or their orthologues in *A. thaliana*, *FSM (FLATENNED SHOOT MERISTEM)/FAS1 (FASCIATA1) and GTE4 (GENERAL TRANSCRIPTION FACTOR GROUP E4) *were already reported to be involved in chromatin remodelling and to affect shoot and root development, meristem differentiation and functioning. The third one encodes a MAP (MICROTUBULE-ASSOCIATED PROTEIN) that may be involved in the control of cell division. Our results support the conclusion that these genes, and the related biological processes, are likely involved in crown root differentiation and are under the control of CRL1.

## Methods

### Plant material and growth conditions

Hulled seeds of wild-type and *crl1 *mutant rice (*Oryza sativa *cultivar Taichung 65) were disinfected and inoculated under sterile conditions in square Petri dishes (Corning, NY, USA) containing half strength Murashige and Skoog (MS) medium (Duchefa Biochemie B.V., The Netherlands) and 0,8% agar type II (Sigma, MO, USA) and were incubated in a vertical position at 26°C and under a 12 h light/12 h dark photoperiod. Stem bases used for transcriptome analysis were collected from 7 day-old plantlets. For auxin treatment, seven day-old axenic seedlings were transferred in flasks containing half strength MS liquid medium, the culture medium was supplemented with 10^-6^M indole acetic acid (IAA) (Sigma) one day after transfer. The *crl1 *mutant is a kind gift of Pr. Makoto Matsuoka (Nagoya University, Japan) [12].

### RNA extraction and preparation

**Figure 1:**
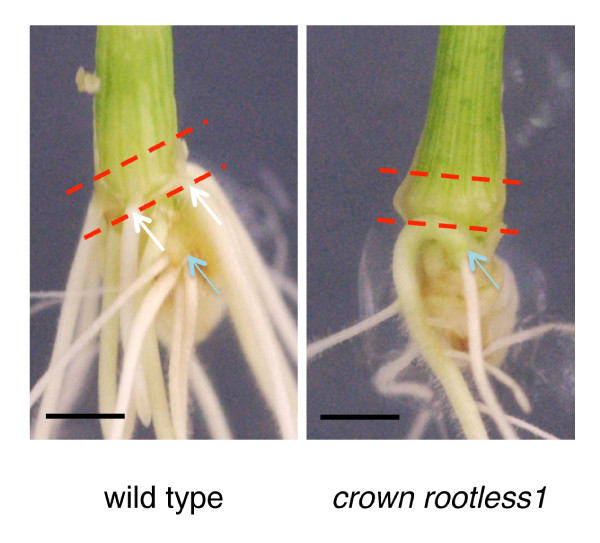
**Portion of stem base collected for transcriptome analysis**. Stem bases of wild type (WT) and *crown rootless1 *(*crl1*) mutant. White arrows show post-embryonic crown roots. Blue arrow shows emergence point of embryonic seminal root. Red dashed lines delimit sampled zone where CR initiate in WT. Scale bar, 4 mm.

For each biological replicate, twenty stem bases (Figure 1) were collected and immediately frozen in liquid nitrogen. RNA were extracted with Plant RNeasy (Qiagen, The Nederlands) and treated on column with Dnase (Qiagen). They were quantified with a NanoQuant at 260 nm wavelength and analysed for quality on a BioAnalyzer 2100 (Agilent, CA, USA). 200 ng of total RNA were used for a single amplification. Antisense RNA strand were then biotynilated according the Affymetrix (Affymetrix, CA, USA) IVT Express protocol.

### Array hybridisation and analysis

All steps were performed according manufacturer's instructions (Affymetrix, CA, USA) unless otherwise stated. Equipments were provided by Affymetrix company. Twelve μg of biotin-labelled antisens RNAs were fragmented and hybridized to *GeneChip Rice Genome Arrays *(Affymetrix) for 16 h at 45°C and 60 rpm in a *Hybridisation Oven 645*. Arrays were washed, labelled with phycoerythrin on the *Fluidic Station 450 *and read with the *Scanner 3000 7G*. Data acquisition was done with the *GeneChip Command Console*. Array pictures were analysed with MAS5 algorithm of the Expression Console software (Affymetrix). Default parameters were applied, global scaling method was used to normalise data (TGT value set at 100). A comparative analysis was carried out for each biological replicate with MAS5 algorithm in the GeneChip Operating software (Affymetrix). Probes with a "Present" Detection Call were kept for subsequent analysis, "Absent" or "Marginal" were rejected. The three biological replicates were compared. Expression of a gene was considered as differentially increased or decreased when its signal ratio was consistently superior to 2 or lower than 0,5 respectively with a p-value P ≤ 0.01 in the three replicates. Orygenes Database http://orygenesdb.cirad.fr/ [16] was used to retrieve gene annotation in rice and in *Arabidopsis *corresponding to selected Affymetrix probes. Microarray data obtained in this study are available in the gene expression omnibus (GEO) database under the reference GSE30818.

### RT-qPCR (Reverse Transcription-quantitative Polymerase Chain Reaction)

cDNA synthesis was performed using RQ DNase I-treated total RNA preparations (see above) and SuperScript III reverse transcriptase kit (Invitrogen). Relative transcript abundance of selected genes (See Additional file 1, Table S1) was determined using the Roche LightCycler 480 system and the LC480 SYBR Green I Master kit (Roche Applied Sciences). The range of primer efficiencies observed for the couples of primers used was comprised between 1.77 and 2. Measurements were taken for three or four biologically independent sets of samples. A technical replicate was performed for each replicate. Expression level of EXP (*Os06g11070*) reference gene was used to normalize gene expression level between the different timepoints [17]. We verified that both in *crl1 *mutant and WT, the *Ct *value of the EXP gene remained stable at different times after auxin treatment and was comprised for different independent biological and technical repetition of the experiment between 27.5 and 29. In addition, LightCycler melting curves were obtained for the reactions, revealing single peak melting curves for all amplification products. The amplification data were analysed using the second derivative maximum method, and resulting Cp values were converted into relative expression values using the comparative Ct method [18]. Mean values of expression levels obtained from different biological repetitions were statistically compared using a Student's t-test.

## Results and discussion

### Transcript profiling of *crownrootless1 *stem base

**Figure 2:**
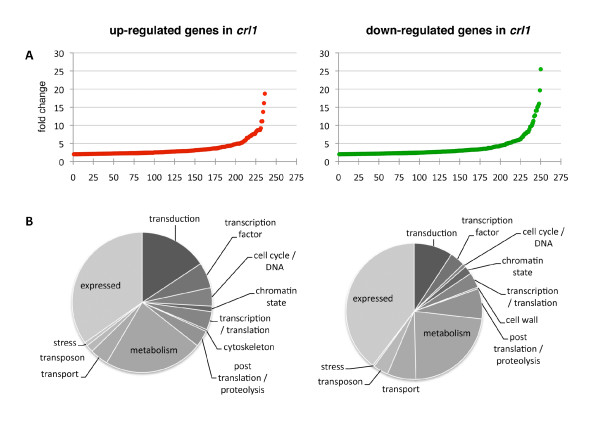
**Transcript profiling of stem bases of *crl1 *mutant**. (A) Fold change of each of the 236 up-regulated and 250 down-regulated genes in the stem bases of *crl1 *mutant relative to the WT. Each abscissa unit corresponds to a unique gene, red points show up-regulated genes, green points show down-regulated genes. (B) Distribution in functional categories of up-regulated (left) and down-regulated (right) genes in *crl1 *relative to WT.

Total RNAs were extracted from WT and *crl1 *stem bases of 7 day-old seedlings. The sampled zone was located just above the zone of emergence of CR in the WT, which correspond to the zone where CR primordia differentiate (Figure 1). RNAs were amplified, labelled and hybridised on GeneChip Rice Genome Arrays (Affymetrix). Transcript profiling of three independent biological replicates obtained from WT and *crl1 *were compared. Only genes exhibiting a significant induction or repression in the three replicates were selected for further analysis. We identified 250 and 236 genes down- and up-regulated respectively at least twofold in the *crl1 *mutant (p-value, P ≤ 0.01). In both groups, about 200 genes were differentially regulated less than 5-fold on average. About 10% of the genes were differentially regulated more than 5-fold in *crownrootless1 *relative to wild-type (Figure 2A). Genes were annotated automatically with function prediction in rice and *Arabidopsis *and categorised manually according to their putative molecular function (Figure 2B). About one third of differentially regulated genes had no predicted function and about one quarter was involved in metabolism. Most of the up-regulated genes in *crl1 *(UPIC) fell into transduction, transcription factor and cell cycle/DNA categories whereas down-regulated genes in *crl1 *(DOIC) were rather distributed in transduction, post-translation/proteolysis and transport categories (Figure 2B).

**Figure 3:**
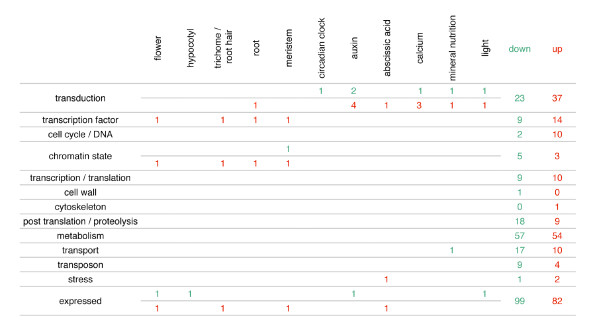
**Genes with a physiological or developmental identified function**. Functional categories are indicated in rows and physiological and developmental processes in columns. Down regulated genes are numbered in green and up regulated in red. "Down" and "Up" columns indicate total gene number for each functional category. Some genes are involved in several processes.

For all genes, and the most similar genes in *Arabidopsis*, we searched for published data relative to their characterised functions. The rice genome sequence has been available since 2005 [19]. Despite an important effort of the scientific community to assign to each gene a function, very few genes have been functionally characterised yet. Precise information was found for only 32 of the 486 genes identified, mostly in reference to a known function of the *Arabidopsis *ortholog (Figure 3). Interestingly, most of these genes had an assigned function related to signal transduction in particular auxin or to gene expression regulation (transcription factors, chromatin remodelling factors) associated with root development or meristem differentiation and functioning.

### Auxin-induced *CRL1 *expression was used to determine *CRL1-*dependent auxin-response genes

**Figure 4:**
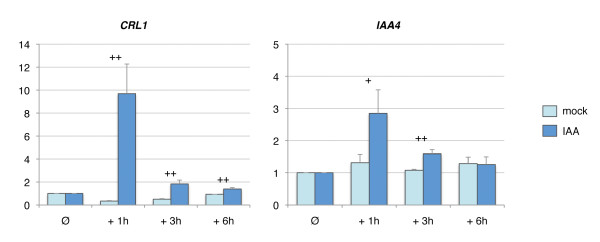
***CRL1 *expression is induced by auxin within 1 hour**. Time course of transcription of *CRL1 *and *IAA4 *genes in response to mock or IAA treatment (0, 1, 3 or 6 hours) in WT and *crl1 *mutant assayed by RT-qPCR. Y-axis indicates the relative expression level at each timepoint relative to timepoint "Ø" (before treatment). IAA4: early-auxin responsive gene, control of IAA treatment. h, hour after treatment. Values represent the mean obtained from 3 independent auxin treatment experiments, bars represent the standard deviation. Mean values were compared between WT and *crl1 *mutant for each time point. A significant difference was noted with one (p-value < 0.1) or two plus signs (p-value < 0.05).

In order to determine how the genes identified by transcript profiling operate in the gene regulatory network downstream of CRL1, we took advantage of the known transcriptional regulation of *CRL1 *by auxin. Auxin (Indole Acetic Acid, IAA) activates *CRL1 *expression within one hour following exogenous treatment (Figure 4). As CRL1 is a transcriptional activator (our unpublished results), we anticipated that expression of CRL1 target genes would increase between 2 and 6 hours following auxin treatment in WT plants, but not in *crl1 *plants. We selected a subset of 47 genes, including most of the 32 genes previously mentioned. These genes were chosen according to a differential regulation in *crl1 *mutant relative to wild-type, their molecular function or their putative role in a developmental process based on available literature data. Expression kinetics of the selected genes in response to IAA treatment (0, 1, 3 or 6 hours) in WT and *crl1 *stem base was measured by RT-qPCR.

As expected, *CRL1 *expression was highly induced 1 hour following auxin treatment and was consistently observed among all the biological replicates with a magnitude ranging between 7 and 12-fold. *CRL1 *expression level decreased following one hour, as a probable result of a negative feedback loop. Expression of *OsIAA4 *(*INDOLE ACETIC ACID4*), an early auxin-responsive gene, was induced in the same time frame than *CRL1 *thereby confirming the efficiency of the auxin treatment [12,20].

### Auxin homeostasis may be altered in *crl1 *stem base

**Figure 5:**
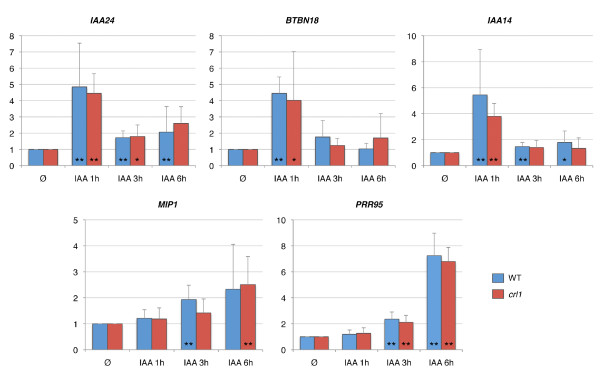
***CRL1-*independent auxin-responsive genes expression profiles**. Time course of transcription of selected genes in response to IAA treatment (0, 1, 3 or 6 hours) in WT and *crl1 *mutant stem bases assayed by RT-qPCR. Y-axis indicates the relative expression level at each timepoint relative to timepoint "Ø" (before treatment). Values represent the mean obtained from 3 independent auxin treatment experiments (blue, WT; red, *crl1 *mutant), bars represent the standard deviation. Mean values obtained at the different timepoints were compared with the corresponding t0 (Ø) mean value. Significant differences were noted with one (p-value < 0.1) or two asterisks (p-value < 0.05). Mean values were compared between WT and *crl1 *mutant for each timepoint, revealing no significant difference.

AUX/IAA genes encode regulatory proteins involved in auxin signal transduction that interact with ARF TF and repress their activity in absence of auxin [21]. TIR1-dependent auxin perception leads to the degradation of AUX/IAA via the ubiquitin pathway and to the regulation of expression of auxin-responsive genes by ARF. This mechanism participates in the regulation of LR differentiation in *Arabidopsis *involving IAA14-mediated ARF7 and ARF19 repression [3,15,22]. In rice, OsARF16 is able to bind *in vitro *an auxin response element in the *CRL1 *promoter. Moreover the expression in rice of a mutated form of OsIAA31 altered in its ubiquitination site causes the inhibition of the induction of *CRL1 *expression by auxin [12] which results in a reduced number of CR [23]. Here we found that *OsIAA14 *was DOIC and that *OsIAA11 *and *OsIAA24 *were UPIC. *OsIAA11 *belongs to the same expression cluster than *OsIAA31 *and is specifically expressed in differentiated roots and stems [20]. *OsIAA14 *is mostly expressed in plumule and floral organs whereas *OsIAA24 *is expressed in various organs including radicle or root [20]. Further RT-qPCR analysis showed that *OsIAA14 *and *OsIAA24 *were early auxin responsive genes both in WT and *crl1 *stem bases (Figure 5). This indicates that their regulation by auxin does not require *CRL1*. It is likely that the absence of root meristem in the stem base of *crl1 *mutant modifies the auxin balance and results in differential regulation of auxin regulated genes independently of *CRL1*. This also holds true for the DOIC *Os09g09370 *gene which encodes OsBTBN18 (Bric-a-Brac, Tramtrack, Broad Complex BTB domain with non-phototropic hypocotyl 3 NPH3 domain) a protein presenting homology with NPH3 family proteins. In *Arabidopsis *the *NPY1 *(*NAKED PINS IN YUC MUTANTS1) *gene belongs to the *NPH3 *gene family and regulates auxin-mediated plant development [24]. *NPY *genes are highly expressed in root tips and contribute to root gravitropism response [25]. Further expression analysis showed that *Os09g09370 *is an early auxin responsive gene but this response to auxin did not require *CRL1 *(Figure 5). In this study we also characterized two late auxin-responsive genes regulated independently of *CRL1: OsMIP1 *(*MADS*-*BOX INTERACTING PROTEIN1*) *and OsPRR95 *(*PSEUDO RESPONSE REGULATOR95*). The DOIC *OsMIP1 *(*Os03g55890*) gene is very similar to *Antirrhinum ternary complex factor MIP1 *gene. MIP1 interacts with MADS-box TF involved in meristem determination during floral transition [26]. The DOIC *OsPRR95 *(*Os09g36220*) gene is orthologous to the *Arabidopsis APRR5 *gene belonging to the *APRR1/TOC1 *(*TIMING OF CAB EXPRESSION 1*) quintet that participates in the circadian regulation of numerous genes notably involved in flowering time and light response [27,28]. The latter results suggest that auxin-regulated genes involved in the control of meristem functioning are differentially regulated in the *crl1 *mutant, but are not under the direct control of *CRL1*.

### Expression of nutrient and water status related genes varies in *crl1 *stem base

The absence of CR in the *crl1 *mutant may also modify the nutrient and water status of the plant. We further analysed the differential expression pattern of genes related to these statuses. Genes involved in mineral homeostasis and in the response to water deficit indeed appear to be differentially regulated in *crl1 *(Figure 3 and Table 1). The DOIC *Os06g03860 *gene encodes a protein orthologous to AtSPX2 (SPX: SYG1/Pho81/XPR1: SYG1, suppressor of yeast gpal; Pho81, CDK inhibitor in yeast PHO pathway; XPR1, xenotropic and polytropic retrovirus receptor). In *Arabidopsis*, AtSPX2 with AtSPX1, AtSPX3 and AtSPX4 are involved in phosphate (P) homeostasis [29]. In rice *OsSPX1 *is a negative regulator of P uptake but the function of the other ones is still unknown [30]. Other genes putatively involved in ion transport such as *Os02g57240 *that encodes the KOB1 (K^+ ^CHANNEL BETA SUB-UNIT1) have been found to be differentially regulated between WT and *crl1 *[31].

**Table 1:** Selected genes for expression analysis by RT-qPCR in response to auxin

	TIGR Id	FC	Annotation	BBMH Arabidopsis	Annotation	References
**UPIC (up-regulated in *crl1 *relative to WT)**						

	Os05g43820	11,11	ras-related protein	At4g28950	ROP9 (RHO-RELATED PROTEIN FROM PLANTS 9)	[54]

	Os08g02490	8,64	AT hook motif domain containing protein	At4g12080	DNA-binding family protein	

**O**	Os02g24740	8,14	OsSAUR9 - Auxin-responsive SAUR gene family member	At4g34760	auxin-responsive family protein	[55,56]

	Os03g51580	6,89	helix-loop-helix DNA-binding domain containing protein	At2g22750	basic helix-loop-helix (bHLH) family protein	

**O**	Os07g22534	5,78	WD domainG-beta repeat domain containing protein	At3g49180	RID3 (ROOT INITIATION DEFECTIVE 3); nucleotide binding	[40,41]

	Os02g02600	5,06	serine/threonine-protein kinase Cx32chloroplast precursor	At2g17220	protein kinase	

**O**	Os12g41900	4,94	SET domain containing protein	At5g42400	SDG25 (SET DOMAIN PROTEIN 25)/ATXR7/MDH9	[46]

**O**	Os02g45810	4,72	WD domainG-beta repeat domain containing protein	At5g24520	TTG1 (TRANSPARENT TESTA GLABRA 1)	[57]

**O**	Os03g20720	3,85	GTPase-activating protein	At1g08680	ZIGA4 (ARF GAP-like zinc finger-containing protein ZiGA4)	[58]

**O**	Os08g40560	3,32	ZOS8-11 - C2H2 zinc finger protein	At2g27100	SERRATE (SE)	[44]

**O**	Os06g05350	2,96	whirly transcription factor domain containing protein	At2g02740	WHY3 (WHIRLY 3); DNA binding; PTAC11	[59]

**O**	Os01g62760	2,88	protein phosphatase 2C	At5g59220	protein phosphatase 2CA	[73,74]

**O**	Os05g14550	2,86	Phosphatidylinositol kinase and FAT containing domain protein	At1g50030	TOR (TARGET OF RAPAMYCIN)	[60]

**O**	Os12g06610	2,54	nucleolar complex protein 2	At3g55510	RBL (REBELOTE)	[45]

**O**	Os12g01140	2,39	ACG kinases include homologs to PKAPKG and PKC	At3g45780	PHOT1 (PHOTOTROPIN 1); protein serine/threonine kinase	[72]

	Os01g49160	2,33	MYB family transcription factor			

**O**	Os05g41070	2,31	bZIP transcription factor	At3g56850	AREB3 (ABA-RESPONSIVE ELEMENT BINDING PROTEIN 3)	[33]

**A O**	Os07g08460	2,30	OsIAA24 - Auxin-responsive Aux/IAA gene family member			[20,61]

**O**	Os03g43890	2,24	WD domain G-beta repeat domain containing protein	At5g58230	MSI1 (MULTICOPY SUPRESSOR OF IRA1)	[62]

	Os01g69850	2,13	OsMADS65 - MADS-box family gene with MIKC type-box			

	Os06g49510	2,06	zinc knuckle family protein	At4g19190	zinc knuckle (CCHC-type) family protein	[63]

	Os07g47820	2,05	acyl-CoA dehydrogenase family member 10	At3g06810	IBR3 (IBA-RESPONSE 3)	[64]

**O**	Os03g43400	2,05	OsIAA11 - Auxin-responsive Aux/IAA gene family member			[20,61]

**O**	Os03g42750	2,02	roothairless 1	At1g47550	AtSec3a (Exocyst complex)	

**DOIC (down-regulated in *crl1 *relative to WT)**						

	Os03g24930	25,48	tyrosine protein kinase domain containing protein	At1g61590	protein kinase	[65]

	Os03g18810	15,00	ll-Diaminopimelate Aminotransferase	At4g33680	AGD2 (ABERRANT GROWTH AND DEATH 2); aminotransferase	[66]

	Os11g11790	8,16	NBS-LRR type disease resistance protein			

**A O**	Os03g55890	8,14	ternary complex factor MIP1	At5g66600	unknown protein	[26]

	Os03g07450	4,24	HOX21 homeobox associated leucine zipper	At1g69780	ATHB13	[67]

**A O**	Os09g36220	3,60	OsPRR95 - response regulator receiver domain containing protein	At5g24470	APRR5 (ARABIDOPSIS PSEUDO-RESPONSE REGULATOR 5)	[68]

	Os08g41340	3,10	ras-related protein	At2g31680	AtRABA5d (Arabidopsis Rab GTPase homolog A5d)	

	Os09g38980	2,81	T-complex protein	At5g18820	EMB3007 (embryo defective 3007)	

**A O**	Os09g09370	2,64	BTBN18 - BTB domain with non-phototropic hypocotyl 3 domain	At5g47800	BTB - NPH3 domain	

	Os04g55560	2,62	AP2 domain containing protein	At4g36920	PLETHORA-like transcription factor	

**CA O**	Os01g67100	2,61	OsFSM, expressed protein	At1g65470	FAS1 (FASCIATA 1)	[48,50]

**CA**	Os08g40620	2,59	rabGAP/TBC domain-containing protein	At4g29950	microtubule-associated protein	

**A O**	Os03g58350	2,40	OsIAA14 - Auxin-responsive Aux/IAA gene family member			[20]

	Os02g08310	2,31	Tubby-like protein 4	At1g16070	AtTLP8 (TUBBY LIKE PROTEIN 8)	[69]

**O**	Os06g03860	2,28	uncharacterized membrane protein	At4g22990	SPX (SYG1/Pho81/XPR1) domain-containing protein	[29]

	Os01g07630	2,18	BRASSINOSTEROID INSENSITIVE 1-associated receptor kinase	At1g60800	NIK3 (NSP-INTERACTING KINASE 3)	

	Os07g04700	2,16	MYB family transcription factor	At3g18100	MYB4R1 (myb domain protein 4R1)	

**O**	Os12g37780	2,13	tetratricopeptide repeat domain containing protein	At2g43040	NPG1 (no pollen germination 1); calmodulin binding	[70]

**CA**	Os02g15220	2,13	bromodomain containing protein, expressed	At1g06230	GTE4 (GLOBAL TRANSCRIPTION FACTOR GROUP E 4)	[51]

	Os04g26850	2,10	SAD2			[71]

	Os10g37640	2,04	HIT zinc finger domain containing protein	At5g63830	unknown protein	

**O**	Os02g52990	2,04	OsSAUR12 - Auxin-responsive SAUR gene family member			[20,61]

**O**	Os10g23220	2,14	GIL1	At2g45260	unknown protein	

"**O**" a gene mentioned in Figure 3. "**A**" indicates a *CRL1-*independent auxin responsive gene. "**CA**" indicates a *CRL1-*dependent auxin responsive gene. ID; identifier. BBMH; Best Blast Mutual Hit. TIGR; Rice Genome Annotation Resource [52-74].

Several UPIC genes with a function related to osmotic stress or abscissic acid were identified. *Os07g05570 *encodes the OsERD4 (EARLY RESPONSIVE DEHYDRATION4) protein orthologous to *ZmERD4 *that enhances drought and salt tolerance when constitutively expressed in *Arabidopsis *[32]. *Os05g41070 *encodes a bZIP transcription factor homologous to AtAREB3 (ABA-RESPONSIVE ELEMENT BINDING PROTEIN 3) that is involved in abscissic acid signal transduction [33]. The DOIC *Os05g14550 *gene encodes a protein orthologous to the conserved eukaryotic TOR (TARGET OF RAPAMYCIN) kinase that promote cell growth in response to favourable conditions. In *Arabidopsis *AtTOR is involved in the inhibition of root growth in response to osmotic stress and excess of nitrate [34]. The absence of CR of the *crl1 *mutant may influence the nutrient and water status of the plant, and consequently modulate the expression of nutrient and stress related genes.

### Genes related to meristem differentiation are deregulated in *crl1 *and some of them are *CRL1-*dependent auxin responsive genes

**Figure 6:**
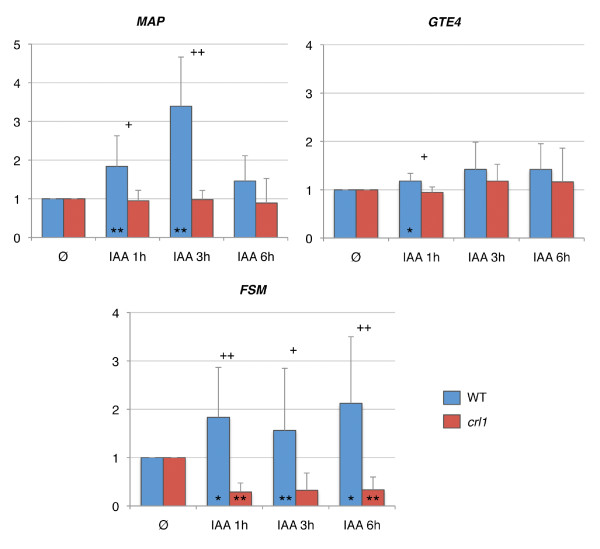
***CRL1-*dependent auxin-responsive genes expression profiles **. Time course of transcription of selected genes in response to IAA treatment (0, 1, 3 or 6 hours) in WT and *crl1 *mutant stem bases assayed by RT-qPCR. Y-axis indicates the relative expression level at each timepoint relative to timepoint "Ø" (before treatment). Value represent the mean obtained from 3 (*MAP*, *GTE4*) to 4 (*FSM*) independent auxin treatment experiments (blue, WT; red, crl1 mutant), bars represent the standard deviation. Mean values obtained at the different timepoints were compared with the corresponding t0 (Ø) mean value. Significant differences were noted with an asterisk (p-value < 0.1) or two asterisks (p-value < 0.05). Mean values were compared between WT and *crl1 *mutant for each time point. A significant difference was noted with one (p-value < 0.1) or two plus signs (p-value < 0.05).

Several genes already identified to regulate processes involved in cell or meristem differentiation are differentially regulated in *crl1*. The DOIC *Os08g40620 *gene encodes a MAP and is a *CRL1-*dependent auxin responsive gene (Figure 6). This suggests that it can be a component of the gene regulatory network directly controlled by *CRL1*. Its function has not been determined yet either in rice or in *Arabidopsis*. Nevertheless the function of other MAP has been characterized in *Arabidopsis*. For example, AtMAP70-5 is involved in the maintenance of cellular polarity and ensures regular extension of plant organs [35]. The *MAP Spiral2 *mutant is defective in directional cell elongation and exhibits right-handed helical growth in longitudinally expanding organs such as the roots [36]. This suggests that the MAP encoded by *Os08g40620 *may be involved in polarized cellular growth during early steps of CR meristem organization regulated by *CRL1*.

Different genes have a function related with root hair or root meristem differentiation. The UPIC *Os03g42750 *gene encodes an Exocyst Complex Component SEC3 family protein orthologous to maize ROOTHAIRLESS1 protein [37,38].

Several UPIC genes encode WD domain G-beta repeat domain containing proteins that can interact with TF or chromatin associated proteins to regulate gene expression and cell or meristem differentiation processes. One of them, *OsTTG1 (TRANSPARENT TESTA GLABRA1) *(*Os02g45810*), is orthologous to *AtTTG1 *that is involved in different epidermis cell differentiation pathways, including root hair differentiation in *Arabidopsis *[39]. *Os07g22534 *is homologous to *AtRID3 *(*ROOT INITIATION DEFECTIVE3*), involved in cell proliferation and in the control of adventitious root initiation in *Arabidopsis *[40,41].

Other genes have a function related to meristem differentiation and functioning. *Os03g43890 *is orthologous to *AtMSI1 *(*MULTICOPY SUPRESSOR OF IRA1*). MSI1-like WD-40 repeat proteins are components of different complexes controlling chromatin dynamics. In plants, MSI1-like proteins are involved in regulatory pathways controlling reproductive meristem transition, embryo and gametophyte development [42,43]. The UPIC *Os08g40560 *gene encodes a putative C2H2 zinc finger protein and is homologous to *SE *(*SERRATE*) in *Arabidopsis *which is involved in miRNA gene silencing pathway that participates to coordinate meristem activity and axial patterning [44]. The DOIC *Os12g06610 *gene encodes a nucleolar complex protein 2 orthologous to *AtRBL *(*REBELOTE*) is involved in floral developmental homeostasis [45]. In addition the UPIC *Os12g41900 *gene, orthologous to *AtSDG25 *(*SET DOMAIN GROUP25*), regulates the epigenetic transcriptional activation and contributes to the regulation of *FLC *(*FLOWERING LOCUS C*), a gene involved in flowering induction in the shoot apical meristem [46,47]. These genes are probably not directly regulated by *CRL1*, but their differential expression in *crl1 *suggests that the lack of CR meristem differentiation may also induce fedback regulatory pathway acting on general TF and chromatin regulatory factors known to be involved in cell and meristem differentiation.

The involvement of chromatin remodelling factor in CR initiation is supported by the fact that two genes related to this process were found to be DOIC and *CRL1-*dependent auxin responsive genes (Figure 6). The gene *OsFSM *(*FLATTENED SHOOT MERISTEM*) (*Os01g67100*) encodes a component of the CAF1 (CHROMATIN ASSEMBLY FACTOR1) complex and is orthologous to *FAS1 *(*FASCIATA1*) in *Arabidopsis *[48-50]. *FAS1 *is involved in the regulation of both shoot and root development regulation. *FAS1 *is strongly expressed in root tips and contributes to the maintenance of *SCARECROW *expression pattern which is essential to root meristem maintenance and root radial differentiation [48]. The rice *fsm *mutant exhibits an altered development including a reduced number of CR, a reduced seminal root growth and strong defects in root meristem structure. *OsFSM *is also expressed in root tips [50]. The other DOIC and *CRL1-*dependent auxin responsive gene (*Os02g15220*) encodes a bromodomain-containing protein homologous to the *Arabidopsis *GTE4. *GTE4 *is involved in the maintenance of the mitotic cell cycle and may be related to chromatin remodelling. In *gte4 *mutants, root development is delayed and the number of lateral root is reduced. In addition, in this mutant, a partial loss of identity is observed for quiescent centre cells and the division pattern of initial cells is disrupted [51]. *FSM *and *GTE4 *play important roles in the control of root meristem differentiation and maintenance. The differential expression of *FSM *and *OsGTE4 *in response to auxin in *crl1 *mutant versus WT suggests that these genes are early CRL1 target genes and stress that chromatin structure modulation and cell cycle regulation may be essential parameters in early steps of CR meristem differentiation in rice.

## Conclusions

### First hypotheses toward the arrangement of the crown root differentiation gene regulatory network controlled by *CRL1*

The differential transcriptome analysis of *crl1 *versus WT allowed the identification of a set of genes differentially regulated in *crl1 *stem base. Further RT-qPCR analysis of the response to auxin of a subset of genes in WT and *crl1 *was used to determine their dependence to *CRL1*. Some genes such as *OsIAA *and *NPH3*-*like*, which are involved in auxin response and auxin-mediated plant development are *CRL1*-independent auxin responsive genes. It is likely that the differential regulation of these genes in *crl1 *versus WT results from the absence of CR meristems that may modify the auxin balance in the *crl1 *mutant stem base tissues. The absence of CR in *crl1 *may also modify the nutrient and water status of the plant and result in the differential expression of genes involved in nutrient uptake or water stress response. Similarly the absence of CR differentiation in *crl1 *may deregulate the expression of genes involved in the control of meristem differentiation and cell division.

Three genes were found to be *CRL1*-dependent auxin responsive genes. They are likely directly regulated by CRL1. This concern *Os08g40620 *which encodes a microtubule-associated protein (MAP) that could be involved in asymmetric control of cell division during early steps of CR meristem differentiation, and the two genes *FSM *and *GTE4 *that encode chromatin remodelling and cell cycle regulation factors known to be involved in rice or *Arabidopsis *on the proper cell division patterning and maintenance of the root meristem.

**Figure 7:**
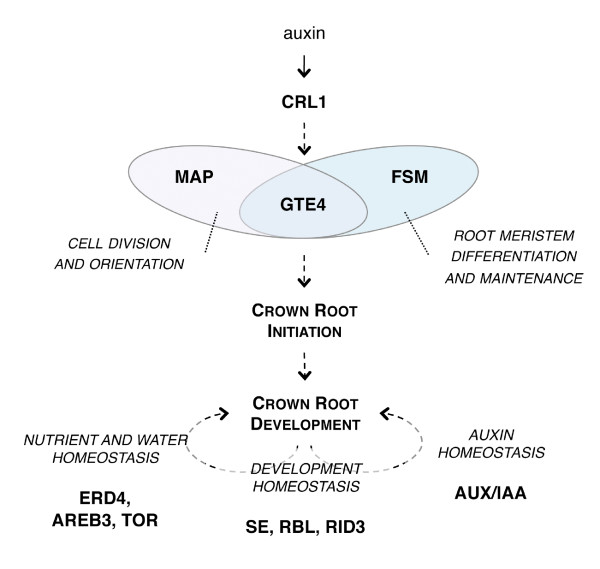
**Hypothetical gene regulatory network controlled by *CRL1 *integrating key genes identified in this study**. Names shown in bold indicate rice genes or their homologues in *Arabidopsis*. Dashed lines indicate hypothetical causal relationships.

Figure 7 summarizes these hypotheses. Among all the genes that we have identified to be misregulated in the *crl1 *mutant, other *CRL1*-dependent auxin responsive genes remain to be identified. Their involvement in CR initiation and development will be further investigated with corresponding insertion mutants.

## Authors' contributions

YC designed the research, performed the experiments and drafted the manuscript. MB, TVAL, MP participated in the RT-qPCR analysis. EG helped in drafting the manuscript. PG designed and coordinated the research and drafted the manuscript. All authors read and approved the final manuscript.

## Supplementary Material

Additional file 1 **Genes analysed and primers used for RT-qPCR**. Primers used for RT-qPCR expression analysis of the 47 genes presented in Table 1 and of *EXP *gene. Click here for file
